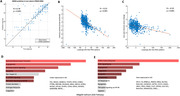# Whole‐blood RNA from different cohorts and profiling techniques consistently predicts cognitive performance across Alzheimer's spectrum

**DOI:** 10.1002/alz.092541

**Published:** 2025-01-09

**Authors:** Gleb Bezgin, Quadri Adewale, Nesrine Rahmouni, Jenna Stevenson, Robert Baumeister, Sue‐Jin Lin, Joon Hwan Hong, Lazaro M Sanchez‐Rodriguez, Simon Ducharme, Pedro Rosa‐Neto, Yasser Iturria Medina

**Affiliations:** ^1^ McGill University, Montreal, QC Canada; ^2^ McConnell Brain Imaging Center, Montreal Neurological Institute, McGill University, Montreal, QC Canada; ^3^ Montreal Neurological Institute, McGill University, Montreal, QC Canada

## Abstract

**Background:**

There is a critical need for minimally‐invasive robust peripheral markers of neurodegenerative conditions. Peripheral RNA may be a powerful tool for in‐depth tracking of biological processes in AD and related disorders. Here, we combine whole‐blood microarray data from Alzheimer's Disease Neuroimaging Initiative (ADNI; N=743) and RNA‐Seq from Translational Biomarkers in Aging and Dementia (TRIAD; N=77) and Montreal Neurological Institute (MNI; N=33) cohorts to predict cognitive performance across AD spectrum.

**Method:**

Whole‐blood RNA from ADNI was profiled using Affymetrix Human Genome U219 Array (www.affymetrix.com); see https://adni.loni.usc.edu/. Whole‐blood RNA from TRIAD and MNI was sequenced using Illumina‐1.9 and aligned to the genome using kallisto (https://pachterlab.github.io/kallisto/); the obtained transcript abundances were brought to gene space using tximport (https://bioconductor.org/packages/release/bioc/html/tximport.html). Microarray and RNA‐Seq data were subjected to BoxCox transformations and harmonized (https://github.com/Jfortin1/ComBatHarmonization), involving study/center correction, preserving variance associated with age, sex, education, Mini Mental State Examination (MMSE) and diagnosis. Regression learning analysis using fine decision tree was performed to evaluate relationship between blood RNA as predictor and MMSE as response variable. The model was trained on ADNI and tested on TRIAD+MNI. To discover over‐ and under‐expressed genes and the subject‐wise AD‐like gene expression patterns, we performed Partial Least Squares (PLS) analysis.

**Result:**

In both the training (ADNI) and independent test (TRIAD+MNI) groups, we observed (Figure 1A) a strong correspondence between real and RNA‐based predicted MMSE values. This supports the blood RNA's capacity to accurately predict cognitive performance across the AD spectrum. PLS loadings significantly correlated (p<0.001) with both memory (Figure 1B) and two‐year longitudinal memory slopes (Figure 1C; includes composite scores for ADNI and MNI, and Rey Auditory Verbal Learning scores for TRIAD). Biological pathways related to the most under‐expressed (Figure 1D) and over‐expressed (Figure 1E) genes included inflammatory response, apoptosis and angiogenesis as top pathways.

**Conclusion:**

We verified the predictive power of whole‐blood gene expression to capture AD cognitive impairment severity, using independent populations, different profiling techniques, and advanced machine‐learning. These results support the feasibility of using minimally invasive blood RNA towards tracking AD progression and potential treatment effects. Among the discovered molecular predictors, the overexpression of inflammatory response is particularly aligned with recent studies proposing neuroinflammation as a hallmark of AD.